# High Temperature High Sensitivity Multipoint Sensing System Based on Three Cascade Mach–Zehnder Interferometers

**DOI:** 10.3390/s18082688

**Published:** 2018-08-16

**Authors:** Na Zhao, Qijing Lin, Zhuangde Jiang, Kun Yao, Bian Tian, Xudong Fang, Peng Shi, Zhongkai Zhang

**Affiliations:** 1 State Key Laboratory for Manufacturing Systems Engineering, Xi’an Jiaotong University, Xi’an 710049, China; zn2015@stu.xjtu.edu.cn (N.Z.); zdjiang@mail.xjtu.edu.cn (Z.J.); vinsent@stu.xjtu.edu.cn (K.Y.); t.b12@mail.xjtu.edu.cn (B.T.); dongfangshuo30@xjtu.edu.cn (X.F.); z.zhongkai@stu.xjtu.edu.cn (Z.Z.); 2 Collaborative Innovation Center of High-End Manufacturing Equipment, Xi’an Jiaotong University, Xi’an 710054, China; 3 State Key Laboratory of Mechanical System and Vibration, Shanghai Jiao Tong University, Shanghai 200240, China; 4 State Key Laboratory of Fluid Power and Mechatronic Systems, Zhejiang University, Hanzhou 310027, China; 5 Electronic Materials Research Laboratory, Key Laboratory of the Ministry of Education & International Center for Dielectric Research, School of Electronic and Information Engineering, Xi’an Jiaotong University, Xi’an 710049, China; spxjy@mail.xjtu.edu.cn

**Keywords:** high temperature sensing, cascade, Mach–Zehnder interferometer, fiber bitapers

## Abstract

A temperature multipoint sensing system based on three cascade Mach–Zehnder interferometers (MZIs) is introduced. The MZIs with different lengths are fabricated based on waist-enlarged fiber bitapers. The fast Fourier transformation is applied to the overlapping transmission spectrum and the corresponding interference spectra can be obtained via the cascaded frequency spectrum based on the inverse Fourier transformation. By analyzing the drift of interference spectra, the temperature response sensitivities of 0.063 nm/°C, 0.071 nm/°C, and 0.059 nm/°C in different furnaces can be detected from room temperature up to 1000 °C, and the temperature response at different regions can be measured through the sensitivity matrix equation. These results demonstrate feasibility of multipoint measurement, which also support that the temperature sensing system provides new solution to the MZI cascade problem.

## 1. Introduction

Multipoint temperature measurement using optical fiber sensors has been extensively applied in various industrial fields, such as oil, gas, aircraft engine, and rail traffic, etc. Compared with commonly used electric sensors [1,2], optical fiber sensors [3,4,5,6,7,8] have received more attention due to their obvious advantages, including high accuracy, easy fabrication, compactness, immunity to electromagnetic interference, and adaptation to complex environments. The multipoint measurement connects sensors at different regions to one optical fiber, which can greatly simplify the sensing system. 

Optical fiber sensors based on Raman scattering [9,10,11,12,13,14,15], Brillouin [16,17], and fiber Bragg grating [18,19,20,21,22,23,24,25,26,27,28,29,30,31,32] are three common ways to realize multipoint measurement. Rusen Yan et al. [9] reported a temperature-dependent Raman sensor, and the temperature sensitivities are −0.011 cm^−1^/K and −0.013 cm^−1^/K for different modes in the range from 100 K to 320 K, respectively. Eilam Yalon et al. [15] also developed a temperature-dependent Raman sensor with sensitivity of −0.016 cm^−1^/°C from 25 °C to 450 °C. However, for the temperature-dependent Raman sensor, a nonlinear effect was observed as temperature increased, which means that the temperature measurement was unmeasurable in ultra-high temperature environments. Yi Bao [16] made a sensor using annealed fused-silica single-mode fiber based on the Brillouin optical time domain analysis, and found that the annealing can affect the upper operation temperature and measurement variability. However, the sensitivity curve is not linear. Meanwhile, fiber Bragg grating (FBG), with the advantage of mature fabrication technique, is extensively applied in the field of multipoint temperature measurement. Cheng Zhang et al. [22] fabricated a new multipoint sensing system by connecting no-core fiber and FBGs, and the temperature responses of the two sensors were 10.05 pm/°C and 10.22 pm/°C from 26.4 °C to 100 °C. However, FBG also has some deficiencies, such as that the reflectance spectrum will be erased^25^ due to the fiber grating obtained by ultraviolet engraving. In order to overcome the deficiency, the femtosecond laser was introduced to improve the temperature tolerance. Xueguang Qiao et al. [29] used femtosecond laser side-illumination to manufacture FBG which had a high temperature resistance up to 1100 °C, with nonuniform temperature response sensitivity. However, the low processing efficiency and expensive equipment of femtosecond laser still limit the wide application of this FBG.

Compared with the above sensors, the Mach–Zehnder interferometer (MZI) [33,34,35,36,37,38,39] has its unique advantages suitable for high-temperature sensors, such as ease of manufacture, high temperature sensitivity and thermal stability. Yufeng Zhang et al. [37] made a MZI by sandwiching a short section of suspended-core fiber between single-mode fibers (SMFs), and the system had a higher sensitivity of 53.87 pm/°C from 200 °C to 1000 °C. Di Wu et al. [39] proposed a novel peanut-shaped MZI with a length of 22 mm, whose temperature sensitivity was 46.8 pm/°C from 100 °C to 900 °C. Linh Viet Nguyen et al. [40] manufactured a MZI based on the structure of multimode fiber- single mode fiber-multimode fiber, and its temperature sensitivity was 0.088 nm/°C from 100 °C up to 900 °C. Jing-Jing Zhu et al. [41] made a MZI based on a thin-core fiber whose linear response is 14.8 pm/°C from normal temperature to 850 °C. L. Jiang et al. [42] presented an optical fiber MZI based on microcavity fabricated with a femtosecond laser, and obtained a sensitivity of 109 pm/°C from 500 to 1200 °C. Ying Wang et al. [43] made a MZI with two sensing arms and achieved a temperature sensitivity of 0.046 nm/°C from 100 °C to 1100 °C. Yun Liu et al. [44] designed a MZI with a piece of multimode fiber sandwiched between two single mode fibers, and achieved a sensitivity of 128.6 nm/°C from 400 °C to 850 °C. Yanping Xu et al. [45] summarized the structure of various fiber optic sensors. Especially for the tapered structure, the taper can be used as a good coupling point, and two tapers can be used to form an MZI. However, there was no investigation on high temperature measurement, and there were few related studies on the MZI cascade. To the best of our knowledge, it was found that there were few solutions to the MZI cascade. The multipoint measurement based on MZI is still a difficulty. 

In this paper, we propose a high temperature MZI multipoint sensing system based on multiplexing technology and sensing characteristics. Three MZIs based on waist-enlarged bitapers were fabricated to detect temperature at different furnaces. The fast Fourier transform (FFT) and inverse fast Fourier transform (IFFT) were adopted to study the output spectrum. Three dual-mode interference (DMI) spectra representing core–cladding interferences were extracted from the cascade sensing spectrum, separately, with the pattern analysis method. In addition, the temperature changes at different furnaces were extracted from the transmission spectra based on different IFFT spectra corresponding to the MZIs. Since the sensing system can overcome the limitation of instability, low temperature sensitivity, large temperature detection range, and cascade problems, the MZI cascade system has a wide application potential for monitoring multipoint temperature at different regions.

## 2. Schematic of the Experimental Setup

**Figure 1:**
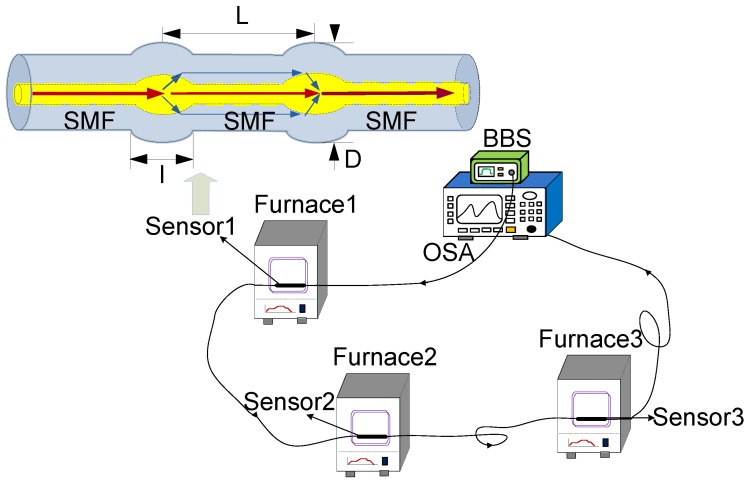
A schematic diagram of the temperature measurement experiment system, and the structure of a sensor used for temperature measurement is magnified.

The schematic diagram of cascading three fiber MZI in the multipoint sensing system is proposed, as shown in Figure 1. It consists of a broad-band light source (BBS), three high-temperature furnaces where the sensors are fixed in, and an optical spectrum analyzer (OSA) used for measuring the final spectrum of the sensing system. The light from the BBS emits into the cascade temperature detection system, and the OSA is used to measure the transmission spectrum of the sensing system with three MZIs connecting in series. 

The multipoint sensing system involves three sensors, as shown in Figure 1. The sensors are used to measure temperatures in different furnaces, and the structure of a sensor based on MZI is amplified. Two bitapers are produced on a SMF as the coupling points, and a section of SMF is used as the sensing arm. The light injected into the first fiber bitaper will excite several modes, which propagate in the optical fiber cladding. A part of light transmits into the fiber core as the fundamental mode, while another part transmits into the optical fiber cladding as the high order mode. Since the modes have different effective refractive index, the optical path difference among different modes occur after transmission through the interfering arm. When the transmission light is transmitted to the second bitaper, the two modes are coupled into the whole spectrum and the interference spectra are obtained through the optical detector. Due to different length of the sensing arm, the MZIs have different optical path variations when the temperature changes. Meanwhile, the optical path differences for different sensors in the multipoint sensor system are superimposed, and the interference fringe of the sensing system is detected. As a result, the temperature at different points can be measured simultaneously through tracing the wavelength of the transmission spectrum.

Due to different length of the sensing arm, the MZIs have different optical path variations when the temperature changes. Meanwhile, the optical path differences for different sensors in the multipoint sensor system are superimposed, and the interference fringe of the sensing system is detected. As a result, the temperature at different points can be measured simultaneously through tracing the wavelength of the transmission spectrum. The fiber used in the experiment is Yofe SMF (Sm28, 8.3 μm/125 μm). A fiber cleaver is was used to get a flat fiber end and the fiber bitaper is was manufactured by ordinary commercial fiber splicing machine. A convenient method to manufacture the fiber bitaper is presented. After being wiped with alcohol, the bare part of the optical fiber was directly fixed in the splicing machine. Then, extrusion welding was carried out through the manual welding mode. In the production preparation process of the fiber bitaper structure, the common fusion program was changed, and the detailed parameters involved were as follows: the electrode discharge intensity was set as 148, the electrode discharge duration time was chosen as 1055 ms, and the pushing distance was set as 128 μm.

## 3. Results and Discussion

**Figure 2:**
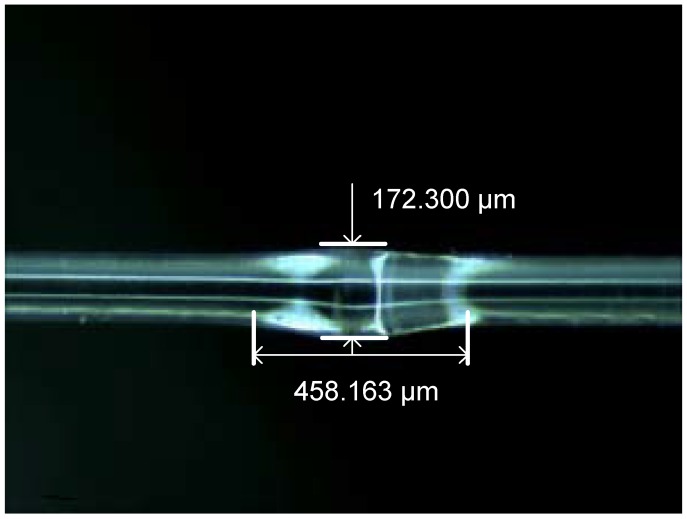
Side view of a waist-enlarged fusion bitaper.

Using the optical microscope, the photograph of the fiber waist-enlarged bitaper was obtained, as shown in Figure 2. The optical fiber bitaper was pushed and formed by increasing the pushing amount in the welding process. In order to get the ideal optical fiber bitaper, the welding procedure was repeatedly modified. The welding effect was mainly affected by the parameters of the discharge intensity, duration time of discharge, and the pushing distance. By the application of welding parameters above, the optical fiber bitaper was made due to the high electrode discharge intensity, long discharge time, and large pushing distance between the fixtures. As a result, the optical fiber diameter at the coupling point was expanded to 160 μm–180 μm, and the length of the coupling point was 400 μm–500 μm. Due to extrusion of the optical fiber by the fixtures, the diameter of the optical fiber was increased, rather than decreased, which contributed to a higher mechanical strength compared with the conventional taper structure.

To simplify the analysis, only two modes are considered in the spectral intensity, as shown in the following formula.

(1) I(λ)=I1(λ)+I2(λ)+2[I1(λ)I2(λ)]12·cos(2πΔneffLλ+ϕ0),

where *I*_1_(*λ*), *I*_2_(*λ*) represent the intensities at the different modes, *λ* represents the wavelength, and *ϕ*_0_ is the initial phase, which is equal to 0. The phase difference Δ*ϕ* can be expressed as

(2) $$\Delta \phi =\frac{2\pi \Delta n_{eff}L}{\lambda }.$$

Considering the interference valley monitored in the experiment, the Δ*ϕ* can be expressed as

(3) Δϕ=(2a+1)·πa=1,2,3……

By combining (2) and (3), we can get the formula

(4) $$\lambda =\frac{2}{2a+1}\cdot \left(\Delta n_{eff}\cdot L\right)a=1,2,3\ldots \ldots$$

From Equation (4), it can be seen that the spectra are related to the length of sensing arm *L* and the effective refractive index difference Δ*n_eff_*. When temperature rises, *L* and Δ*n_eff_* are affected by thermal expansion effect and the thermo-optic effect, which in turn, lead to spectral changes. Moreover, using the Taylor expansion to expand Equation (2), stripe interval Λ can be formulated as

(5) $$\Lambda =\frac{\lambda^{2}}{2\cdot \Delta n_{eff}L}$$

**Figure 3:**
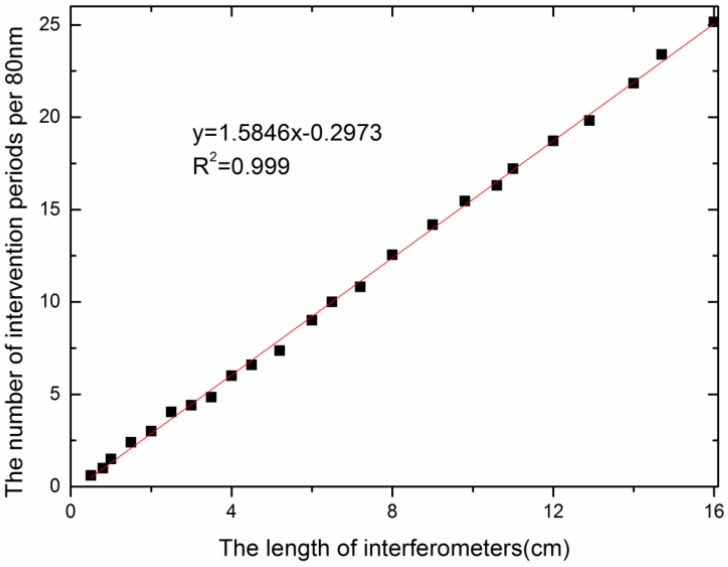
The relationship between the number of intervention periods and the length of sensing arm.

Figure 3 shows the variety of interference period with different sensing arms, which can be analyzed by Equation (5). As the sensing arm length increases, the stripe interval decreases, i.e., the increase of the sensing arm leads to an increase in the number of interference fringes per unit. Among them, the length of the interferometer is represented by *L*, and the number of intervention periods per 80 nm is expressed as *N*. Corresponding to an increase of sensing arm from 0 to 16 cm, the change of the interference period is from 0 to 25 with a good linear correlation coefficient of 0.999 in the wavelength range from 1500 nm to 1580 nm. The above results show that the length of the sensing arm is an important parameter. However, when the distance between the coupling points is large enough, the interference fringes are denser and the interference intensity is weaker. There is no obvious interference when the sensing arm is more than 16 cm. In this circumstance, the sensing effect is extremely weak, which is similar to the optical fiber only used for transmission. Therefore, when different sensors were designed to be cascaded, the distance between two MZIs distributed on the optical fiber is at least more than 16 cm.

**Figure 4:**
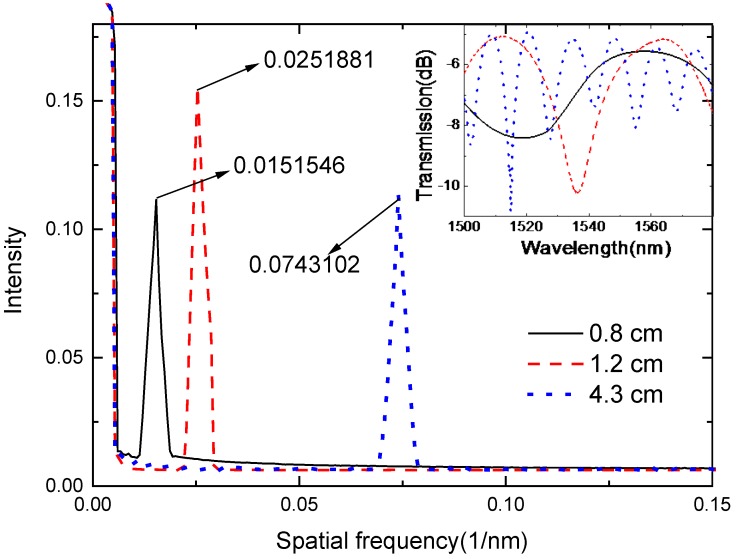
The spatial frequency spectra of Mach–Zehnder interferometers (MZIs), and the inset is the correlated interference spectra with different sensing arms of 0.8 cm, 1.2 cm, and 4.3 cm.

The spatial frequency spectra of the MZIs can be obtained through the transmission spectra. As shown in Figure 4, the selected spectra with a high fringe visibility were extracted to take the FFT, and some high order modes were detected. The results indicate that the dominant peak amplitudes are located at 0.0151546 nm^−1^, 0.0251881 nm^−1^, and 0.0743102 nm^−1^, while the length of the sensing arms are 0.8 cm, 1.2 cm, and 4.3 cm, respectively.

**Figure 5:**
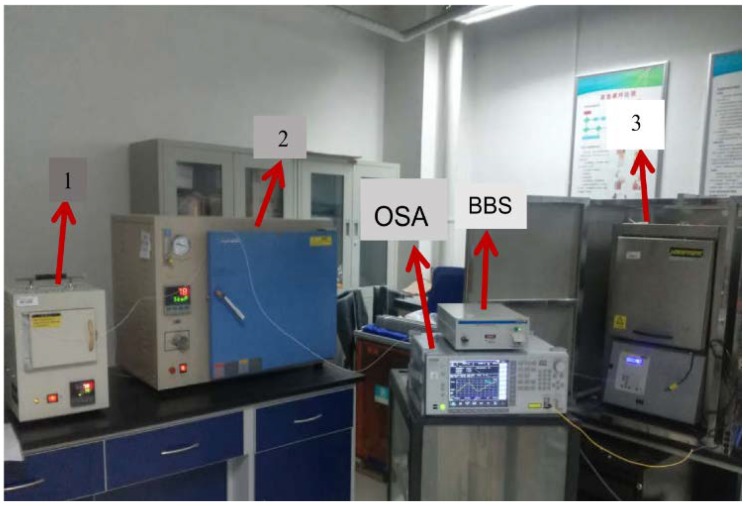
The experimental site of the multipoint temperature sensing system. The light from the broad-band light source (BBS) emits into the cascaded MZIs, and the MZIs are placed in three furnaces, respectively. The final superposition of the three sensors is obtained through the optical spectrum analyzer (OSA).

Figure 5 is the picture of the temperature experiment system, including the bandwidth of the light source (Lightcomm (Shenzhen, China), ASE-CL), which was from 1500 nm to 1580 nm, and the connection fiber was ordinary optical SMF, and the length was about 3 m. The in-fiber MZIs with the sensing arms of 0.8 cm, 1.2 cm, and 4.3 cm were fixed in three furnaces (STAMF-1400, DHG-9145D, and NBD-O1200-60IC), which had a temperature control accuracy of 1 °C, and the temperature interval was set to be 100 °C. The furnaces were heated to more than 300 °C, and then cooled down to room temperature to ensure the coating layer of the fiber was removed. The optical spectrum analyzer (OSA, Anritsu (Atsugi-shi, Japan), MS9740A) with the wavelength resolution of 0.02 nm and the effective measurement range of 600–1700 nm, was used as the final collection device of the spectrum.

**Figure 6:**
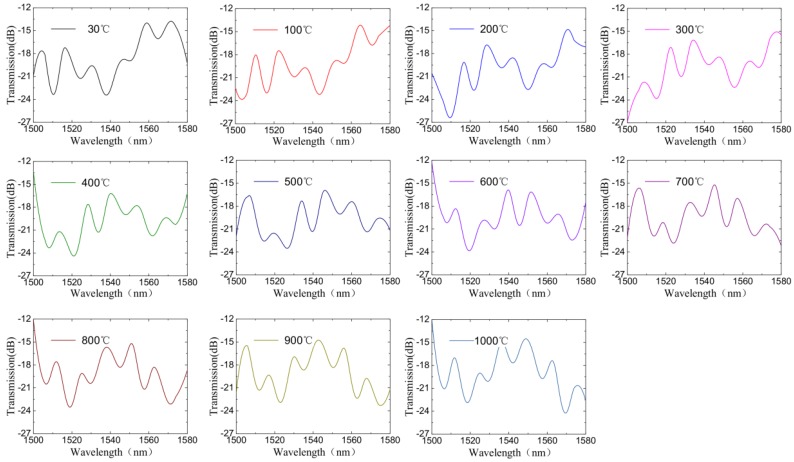
The transmission spectra of the sensing system based on three cascade MZIs at different temperatures.

The sensors were used to monitor the surrounding temperature. The temperature characteristic of the proposed sensing system was also investigated by mounting the sensors in three furnaces. The three furnaces were set to raise the temperature by 100 °C each time, and held for 1 h every time. Due to the limit of the furnace’s maximum temperature, the second furnace was kept at 500 °C after raising the temperature five times. In this experiment, the MZI with a length of 0.8 cm was placed at the first furnace with a temperature range of 30 °C to 1000 °C. The second one with a length of 1.2 cm was placed at the second furnace with a temperature range of 30 °C to 500 °C, and the third one with a length of 4.3 cm was placed at the third furnace with a temperature range of 30 °C to 1000 °C. As shown in Figure 6, the superposition spectrum of the three sensors are obtained through the OSA. Through the obtained transmission spectrum, we analyzed the Fourier frequency spectra and performed IFFT on each frequency spectrum. Accordingly, the waveform drift of each sensor can be demodulated, and the external environment variety of each sensor can be deduced. 

**Figure 7:**
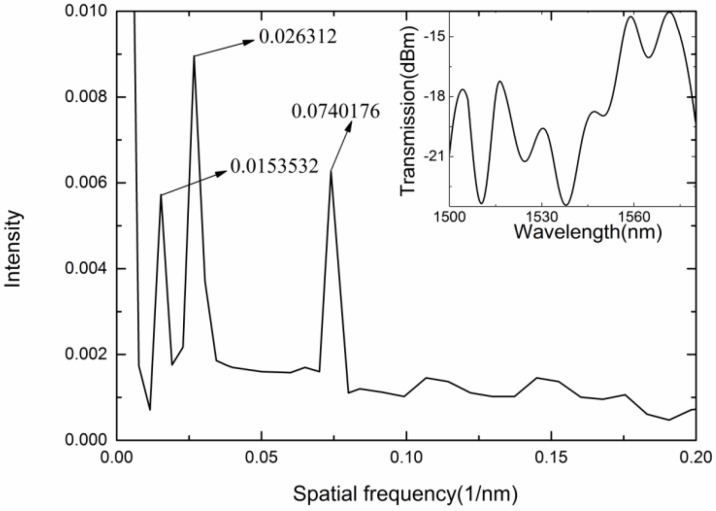
The spatial frequency spectrum of the cascade system and the inset is the corresponding spectrum.

The temperature measurement system cascaded with three MZIs was used to determine the mode order based on FFT. According to FFT results obtained in Figure 7, the interference produced by the three sensors was strong, and the dominant peak amplitudes were located at 0.0153532 nm^−1^, 0.026312 nm^−1^, and 0.0740176 nm^−1^, respectively. This showed that the transmission spectrum of the MZI was dominantly formed by the superposition of three DMI spectra. Compared with Figure 4, there were three main peaks in Figure 7, which corresponded to the FFT peaks when three sensors were measured individually. It could be concluded that superposition of different cosine functions constituted the last spectrum.

For further modal analysis, the central wavelength was extended by Taylor expanding, then the phase *ϕ* could be formulated as:

(6) $$\phi \approx \phi_{0}-\frac{2\pi \Delta \lambda }{\lambda^{2}}\Delta n_{eff}\cdot L,$$

where Δ*λ* is the wavelength difference, *ϕ*_0_ is the initial phase, *λ* is the center wavelength of the interference valley, Δ*n_eff_* is the refractive index difference. A peak in the spatial frequency spectrum corresponds to a sinusoidal interference pattern. If the initial phase equals to 0, the spatial frequency *ξ* [46] can be expressed as

(7) $$\xi =\frac{1}{\lambda^{2}}\Delta n_{eff}\cdot L.$$

**Figure 8:**
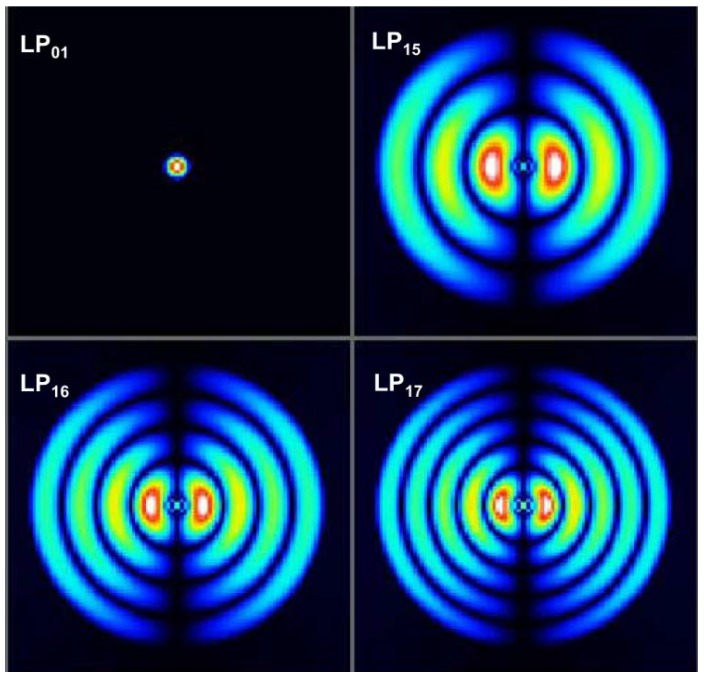
The mode profiles of different modes.

The relationship between Δ*n_eff_* and different modes was obtained using the software of OptiFiber2.0 (Ottawa, ON, Canada), as shown in Table 1. The center wavelength *λ* was 1515.520 nm, and the lengths of MZIs were 0.8 cm, 1.2 cm, and 4.3 cm, respectively. Through the FFT, the spatial frequency *ξ* were 0.0153532 nm^−1^, 0.026312 nm^−1^, and 0.0740176 nm^−1^, respectively. Therefore, the parameter Δ*n_eff_* calculated from Formula (7) were 0.0045514, 0.0052001, and 0.0041355, respectively. They approximately agree with the refractive index difference between core mode LP_01_ and high-order mode LP_16_, LP_17_, and LP_15_, and Figure 8 shows the mode profiles at different modes.

**Figure 9:**
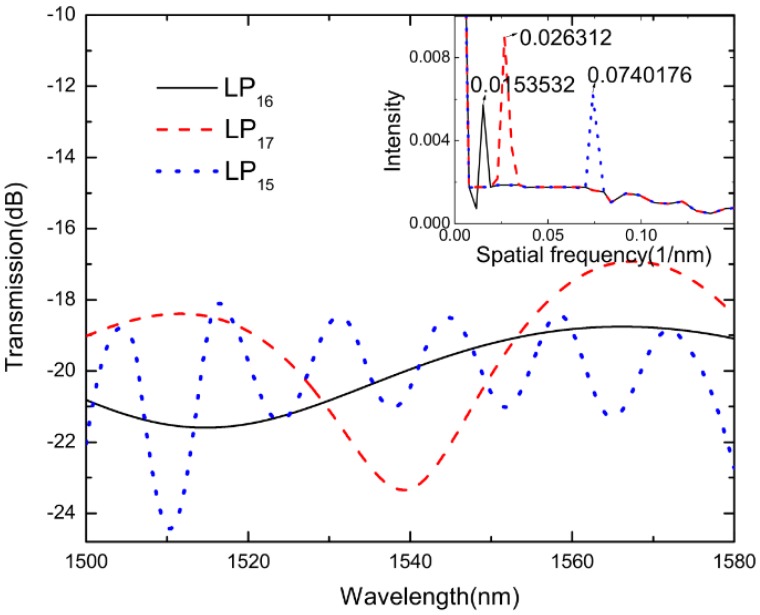
The derived MZI transmission spectra at different furnaces, and the inset shows the corresponding spatial frequency spectra.

According to the principle that any periodic signal can be decomposed into a series of sinusoidal signals, which are related to the specified amplitudes and frequencies, the different sinusoidal signal spectra can be extracted from the cascaded spectrum. The frequency spectrum was used to analyze the characteristics of the sensing system, and an important feature was that the stabilized interfering modes exist with the temperature increase. As shown in Figure 9, we extracted the main peaks in the Fourier spectrum to take the IFFT and obtain the spectra. The temperature variation of one furnace can be obtained from the Fourier spectrum variation based on one of core–cladding interference patterns. 

**Figure 10:**
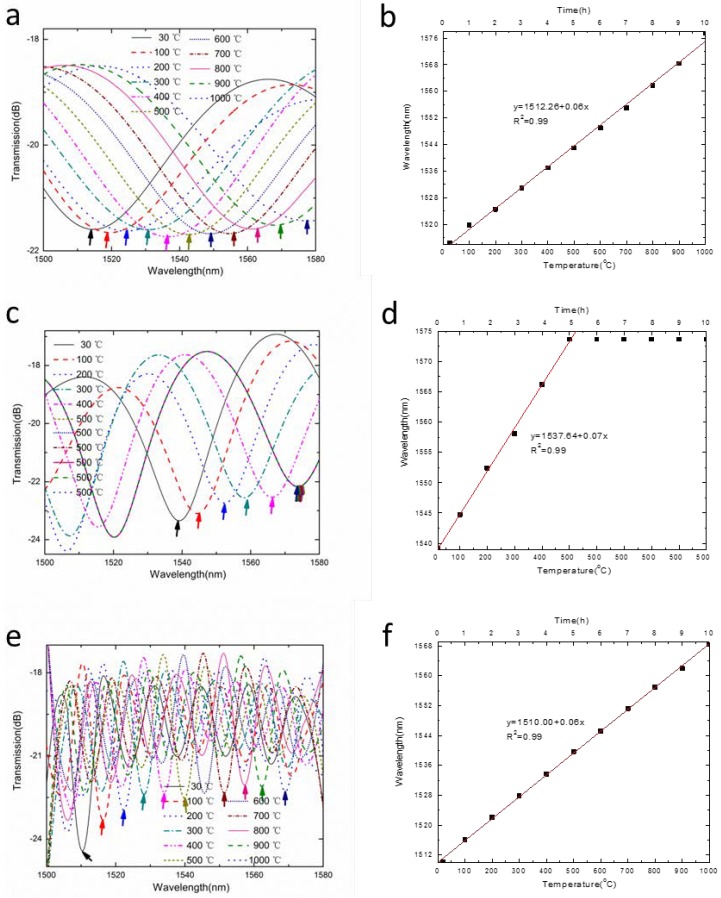
The drift of MZI spectra and corresponding temperature response at different furnaces. (**a**,**b**) correspond to the first furnace; (**c**,**d**) correspond to the second furnace; (**e**,**f**) correspond to the third furnace.

When the external environment changed, the IFFT spectrum corresponding to different measuring points would drift. Specifically, the thermo-optic coefficient of the optical fiber core was higher than the optical fiber cladding, which resulted in the increase of Δ*n_eff_* with the temperature increase. Due to the thermal expansion effect, the length of the optical fiber increased. As a result, the interference spectra of MZIs would drift towards long wavelengths, due to the temperature variation. Figure 10a,b represented the spectral drift corresponding to the MZI at the first furnace. The sensing arm length of MZI was 0.8 cm, and the interference was between LP_01_ and LP_16_. The experiment result showed that the temperature sensitivity was 0.063 nm/°C from 30 °C to 1000 °C with good linearity.

Similarly, we performed IFFT on the second MZI frequency spectra at different temperatures. The temperature sensitivity was obtained. As shown in Figure 10c, they corresponded to the spectral drift of the MZI with the sensing arm length of 1.2 cm based on the interference of LP_01_ and LP_17_, and the spectral temperature response corresponded to the second furnace. When the temperature increased to 500 °C after 5 h, the temperature remained the same for 5 h. For the resonance dip at 1537.642 nm, the temperature sensitivity of MZI was obtained using linear fitting method, as shown in Figure 10d. The temperature sensitivity of the MZI at second furnace was 0.071 nm/°C, and the spectral drift was not obvious during temperature maintenance. Figure 10e,f corresponded to the spectral drift of the MZI, with the sensing arm length of 4.3 cm corresponding to the third furnace based on the interference of LP_01_ and LP_15_. For the resonance dip at 1510.006 nm, the temperature sensitivity of the MZI at third furnace was 0.059 nm/°C with a high linear correlation coefficient of 0.999.

When the temperature of the three furnaces change, the spectra superimposed by three MZIs will drift, which make it possible to simultaneously measure temperature at different points. As the spectrum corresponding to the interference at different temperature detection point can be demodulated simultaneously, the character matrix can be used to represent the temperature response of MZI sensing system. Suppose *λ*^0^ is the initial wavelength, and the wavelength is obtained by the OSA, *T* is the temperature to be measured in the experiment. The wavelength can be expressed as follows:

(8) $$\left[\begin{array}{c} \lambda_{1}\\ \lambda_{2}\\ \lambda_{3} \end{array}\right]=\left[\begin{array}{c} \Delta \lambda_{1}^{0}\\ \Delta \lambda_{2}^{0}\\ \Delta \lambda_{3}^{0} \end{array}\right]+\left[\begin{array}{ccc} k_{1} & 0 & 0\\ 0 & k_{2} & 0\\ 0 & 0 & k_{3} \end{array}\right]\cdot \left[\begin{array}{c} T_{1}\\ T_{2}\\ T_{3} \end{array}\right]$$

By multiplying the reciprocal matrix and combining the Formula (8), the temperature of three furnaces can be obtained as follows:

(9) $$\left[\begin{array}{c} T_{1}\\ T_{2}\\ T_{3} \end{array}\right]=\left[\begin{array}{ccc} {k_{1}}^{-1} & 0 & 0\\ 0 & {k_{2}}^{-1} & 0\\ 0 & 0 & {k_{3}}^{-1} \end{array}\right]\cdot \left[\begin{array}{ccc} k_{1} & 0 & 0\\ 0 & k_{2} & 0\\ 0 & 0 & k_{3} \end{array}\right]\left[\begin{array}{c} T_{1}\\ T_{2}\\ T_{3} \end{array}\right]=\left[\begin{array}{ccc} {k_{1}}^{-1} & 0 & 0\\ 0 & {k_{2}}^{-1} & 0\\ 0 & 0 & {k_{3}}^{-1} \end{array}\right]\cdot \left[\begin{array}{c} \lambda_{1}-\lambda_{1}^{0}\\ \lambda_{2}-\lambda_{2}^{0}\\ \lambda_{3}-\lambda_{3}^{0} \end{array}\right]=[\begin{array}{c} {k_{1}}^{-1}\cdot \left(\lambda_{1}-\lambda_{1}^{0}\right)\\ {k_{2}}^{-1}\cdot \left(\lambda_{2}-\lambda_{2}^{0}\right)\\ {k_{3}}^{-1}\cdot \left(\lambda_{3}-\lambda_{3}^{0}\right) \end{array}]$$

Formula (9) is used to calculate the temperature in three furnaces. In the temperature experiment, the interference valley at the wavelengths of 1512.255 nm, 1537.642 nm, and 1510.006 nm were detected, and the sensitivities of three sensors were 0.063 nm/°C, 0.071 nm/°C, and 0.059 nm/°C, respectively. Therefore, the temperatures at different points in three furnaces can be simultaneously detected with the help of the matrix method. The formula for calculating the temperature is shown in Formula (10).

(10) $$\left[\begin{array}{c} T_{1}\\ T_{2}\\ T_{3} \end{array}\right]=\left[\begin{array}{c} 0.063^{-1}\cdot \left(\lambda_{1}-1512.255\right)\\ 0.071^{-1}\cdot \left(\lambda_{2}-1537.642\right)\\ 0.059^{-1}\cdot \left(\lambda_{3}-1510.006\right) \end{array}\right]$$

## 4. Summary

A temperature sensing system based on fiber bitaper MZI was designed, and the temperature experiment verified the feasibility of multipoint measurement. The FFT and IFFT were applied to the transmission spectra of the MZI multipoint sensing system for temperature measurement. Three different IFFT spectra corresponding to MZIs with different interference lengths were detected, and the temperature changes at different furnaces were extracted from the transmission spectra. The temperature response of each MZI was 0.063 nm/°C from 30 °C to 1000 °C, 0.071 nm/°C from 30 °C to 500 °C, and 0.059 nm/°C from 30 °C to 1000 °C, respectively. In addition, the temperature variations and the corresponding temperature values of every furnace can be detected by applying the sensitivity matrix equation. The system consisting of three MZIs features the advantages of compact, high sensitivity, and large dynamic range for multipoint temperature measurement.

## Figures and Tables

**Table 1:** The values of Δ*n_eff_* between the core mode LP_01_ and a certain high-order mode.

	LP_0*x*_		LP_1*x*_	
X	*n* *_LP_*_0*x*_	Δ*n_eff_*	*n* *_LP_*_1*x*_	Δ*n_eff_*
1	1.4658679	0	1.4630794	0.0027885
2	1.4627498	0.0031181	1.4627169	0.003151
3	1.4625833	0.0032846	1.4625135	0.0033544
4	1.4623038	0.0035641	1.4621959	0.003672
5	1.4619147	0.0039532	1.461768	0.0040999
6	1.4614194	0.0044485	1.4612316	0.0046363
7	1.4608213	0.0050466	1.460588	0.0052799
8	1.4601245	0.0057434	1.459838	0.0060299
9	1.4593324	0.0065355	1.4589826	0.0068853

## Abbreviations

The following abbreviations are used in this manuscript:

FFT	fast Fourier transform
FBG	fiber Bragg grating
MZI	Mach–Zehnder interferometer
SMF	single mode fiber
IFFT	inverse fast Fourier transform
DMI	dual-mode interference
BBS	broad–band light source
OSA	optical spectrum analyzer
